# The Time Course of Inflammatory Biomarkers Following a One-Hour Exercise Bout in Canines: A Pilot Study

**DOI:** 10.3390/ani10030486

**Published:** 2020-03-13

**Authors:** Wendy Pearson, Julia Guazzelli Pezzali, Renan Antunes Donadelli, Ashley Wagner, Preston Buff

**Affiliations:** 1 Department of Animal Biosciences, University of Guelph, Guelph, ON N1G 2W1, Canada; jguazzel@uoguelph.ca (J.G.P.); rantunes@uoguelph.ca (R.A.D.); 2 Probiotech International, Saint-Hyacinthe, QC J2S 8L2, Canada; ashleywagner@probiotech.com; 3 VetDiet, Montreal, QC H1B 1C9, Canada; pbuff@vetdiet.com

**Keywords:** exercise, inflammation, resolvin D1, prostaglandin E_2_, biomarkers, canine

## Abstract

**Simple Summary:**

The purpose of this study is to generate preliminary data on the inflammatory effects of an hour of hunting in dogs. Four basset hounds were set out to find a scent and freely adopted running or walking over wooded terrain for one hour. Blood samples were obtained before exercise and 1, 2, 4, 6, and 10 h after the end of the exercise for analysis of markers of inflammation (prostaglandin E_2_ (PGE_2_), nitric oxide (NO), interleukin 1β (IL-1β)), tumour necrosis factor-α (TNF-α)), and inflammation resolution (resolvin D1 (RvD1)). There was an increase in inflammation one hour after the exercise, shown by a significant increase in PGE_2_. Following the peak, PGE_2_ steadily declined at the same time as RvD1 increased, with RvD1 peaking at six hours. This pilot study provides evidence that dogs that undergo an hour of hunt exercise experience transient inflammation that peaks one hour after the end of exercise; inflammation resolution peaks six hours after the end of exercise. Future studies should seek to further understand the distinct and combined roles of PGE_2_ and RvD1 in dog adaptation to exercise stress.

**Abstract:**

There is little information available to describe the inflammatory consequences of and recovery from moderate-intensity exercise bouts in hunting dogs. The purpose of the current study is to generate pilot data on the appearance and disappearance of biomarkers of inflammation and inflammation resolution following a typical one-hour exercise bout in basset hounds. Four hounds were set out to find a scent and freely adopted running or walking over wooded terrain for approximately one hour. Venous blood samples were obtained before the exercise and at 1, 2, 4, 6, and 10 h following cessation of exercise and were analyzed for biomarkers of inflammation (prostaglandin E_2_ (PGE_2_), nitric oxide (NO), interleukin 1β (IL-1β)) tumour necrosis factor-α (TNF-α)), and inflammation resolution (resolvin D1 (RvD1)). There was an increase in inflammation one hour after the exercise, shown by a significant increase in PGE_2_. Following this peak, PGE_2_ steadily declined at the same time as RvD1 increased, with RvD1 peaking at six hours. This pilot study provides evidence that dogs that undergo an hour of hunt exercise experience transient inflammation that peaks one hour after the end of exercise; inflammation resolution peaks six hours after the end of exercise. Future studies should seek to further understand the distinct and combined roles of PGE_2_ and RvD1 in dog adaptation to exercise stress.

## 1. Introduction

Transient inflammation induced by exercise is a physiological phenomenon that can be observed in several athletic species, including humans [1,2,3], horses [4,5], and dogs [6,7,8]. Pro-inflammatory cytokines, such as interleukin-1 β (IL-1β) [9,10] and tumour necrosis factor alpha (TNF-α) [1,11], transiently increase following a high-intensity exercise bout, as do pro-inflammatory eicosanoids such as prostaglandin E_2_ (PGE_2_) [4,5,12] and reactive oxygen species such as nitric oxide (NO) [4,5]. While it is tempting to characterize this phenomenon as a negative consequence of exercise due to evidence for breakdown of tissues, such as loss of articular glycosaminoglycan [4,5,13], it is becoming increasingly clear that exercise-induced inflammation has a complex and essential role in adaptation (i.e., training) of tissues [14,15], contributing to a net *anti*-inflammatory effect of repeated exercise bouts over time [14,16,17]. Indeed, pharmacological blunting of the inflammatory responses to exercise bouts appears to impair the active resolution of inflammation and consequent adaptation of tissue [18,19]. A candidate molecule for initiation and propagation of inflammation resolution is resolvin D1 (RvD1). RvD1 results from the metabolism of eicosapentaenoic acid (EPA) and docosahexaenoic acid (DHA) and functions to restore normal tissue homeostasis following an inflammatory event [20]. RvD1 production is stimulated, at least in part, by catecholamines [20], which provide a likely explanation for the increase in RvD1 following exercise [12,19].

The sport of hunting wild game with basset hounds originated in France and has long been popularized throughout Europe and North America. Basset hounds are considered to be exceptional hunters because of their high ability to ground scent [21,22]. During the hunt, great distances are travelled when the dogs are searching and following a scent. As a result, the hunting exercise is characterized as an endurance activity in which acute bouts of high-intensity running are interspersed with low-intensity walking over a period of one or more hours. The effects of this type of exercise on biomarkers of inflammation and inflammation resolution were not described in the past. Furthermore, the post-exercise time-course of biomarker appearance and disappearance in dogs undergoing hunt exercise is also unknown; this information is important in order to compare the effects of variables such as diet and pharmaceuticals on post-exercise inflammation and inflammation resolution. In addition, this information can be applied to improve the management of musculoskeletal injury, as well as prevent and treat chronic inflammatory diseases with exercise. Thus, the objective of the current pilot study is to quantify the time-course of systemic biomarkers of inflammation and inflammation resolution within 10 h of a typical bout of hunt exercise in basset hounds. It is hypothesized that a hunt exercise produces transient inflammation, which can be observed through an increase in the plasma levels of pro-inflammatory PGE_2_, NO, IL-1β, and TNFα. Furthermore, the later stage of exercise recovery is hypothesized to be characterized by an increase in plasma levels of the pro-resolving mediator RvD1.

## 2. Materials and Methods

The study protocol was reviewed and approved by the VetDiet Animal Welfare Committee under the guidelines for the Canadian Council on Animal Care (protocol approval number ACC 091119).

All reagents were purchased from Sigma Aldrich, Mississauga (Ontario, Canada), unless otherwise indicated.

### 2.1. Animals

This pilot study was carried out at a private farm located in Lachute (Quebec, Canada). Four (4) client-owned Basset Hounds (three males, one female; 4.5 ± 1.12 years old; 23 ± 3 kg) that live habitually in an indoor kennel (3 × 12 m) with other dogs of the same breed, mixed gender, and similar activity/fitness level were included in the study. Dogs were adapted to one to two hours of daily hunt exercise for a minimum of six months, covering a distance of 2.5–5 km per day. They were fed a commercial diet (Vetdiet^®^ Adult All Breeds Chicken and Rice Formula; Mondou, Montreal QC, Canada) at a rate of 0.4 kg (1400 kcal)/day once per day at 9:00 a.m. to maintain body weight. On the study day, study dogs were grouped with non-study dogs and set out to find scent trails immediately following their morning meal. The dogs were permitted to freely adopt a running or walking regimen over wooden terrain with many natural woodland obstacles, whilst their guide followed on foot. The hunt exercise continued for approximately one hour, over a distance of approximately 2.5 km.

### 2.2. Sample Collection

Blood (2–3 mL) was collected by cephalic or jugular venipuncture using a sterile 22-gauge needle 30 min before (PRE) and 1, 2, 4, 6, and 10 h after completion of the hunt exercise. Immediately after collection, blood samples were transferred to a heparin-coated vacutainer tube, which was gently inverted 8–10 times to ensure the proper mixing of blood with the anticoagulant. Heparinized samples were then transferred to 1.5 mL microcentrifuge tubes and centrifuged (11,000 RPM for 15 min at room temperature). Plasma was transferred to a new 1.5 mL microcentrifuge tube and frozen (−80 °C) until analysis.

### 2.3. Plasma Analysis

All plasma samples were analyzed undiluted. ELISA kits validated for use in canine plasma were purchased from MyBiosource, San Diego (California, USA). For ELISAs, all samples underwent one freeze–thaw cycle and were all analyzed on the same day. After conducting the ELISA tests, plasma samples were refrigerated at 4 °C overnight before being analyzed for NO on the following day.

PGE_2_ (catalogue #MBS735120), RvD1 (catalogue #MBS057451), IL-1β (catalogue #MBS165846), and TNFα (catalogue #MBS9305457) assays were conducted according to kit instructions. Linear regression equations were developed from standard curves on each plate and used to calculate concentrations of biomarkers in each plasma sample.

NO was analyzed using the Griess Reaction. Plasma samples were analyzed as previously described [4]. A linear regression equation was developed from a nitrite standard curve and used to calculate the concentration of NO in each well.

### 2.4. Data Analysis

Data are presented as mean ± standard error (SE), unless otherwise indicated. Data were analyzed using a one-way Repeated Measures ANOVA, with “time” as the fixed effect (SigmaPlot Version 12). When a significant f-ratio was obtained, the Holm–Sidak post-hoc test (all-pairwise and each post-exercise point compared to PRE) was used to identify significantly different means. Significance was accepted at *p* < 0.05.

## 3. Results

### 3.1. PGE_2_

**Figure 1:**
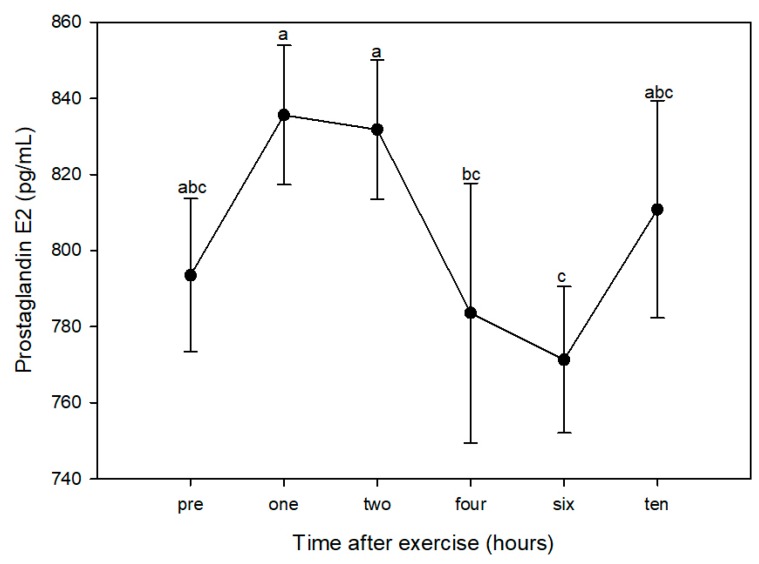
Plasma levels of prostaglandin E_2_ (PGE_2_) in four dogs prior to (PRE) and 1, 2, 4, 6, and 10 h after a one-hour hunt exercise bout. Time points with different letters indicate significantly different PGE_2_ concentrations using the “all-pairwise” comparison procedure (*p* < 0.05).

Using pre-exercise values (793.5 ± 20.1 pg/mL) as the control within the post-hoc test, there was a significant increase in PGE_2_ one hour after exercise (835.6 ± 18.3 pg/mL). PGE_2_ was significantly higher at one and two (831.8 ± 18.3 pg/mL) hours post-exercise than at four (783.6 ± 34.2 pg/mL) and six (771.3 ± 19.2 pg/mL) hours post-exercise (Figure 1).

### 3.2. RvD1

**Figure 2:**
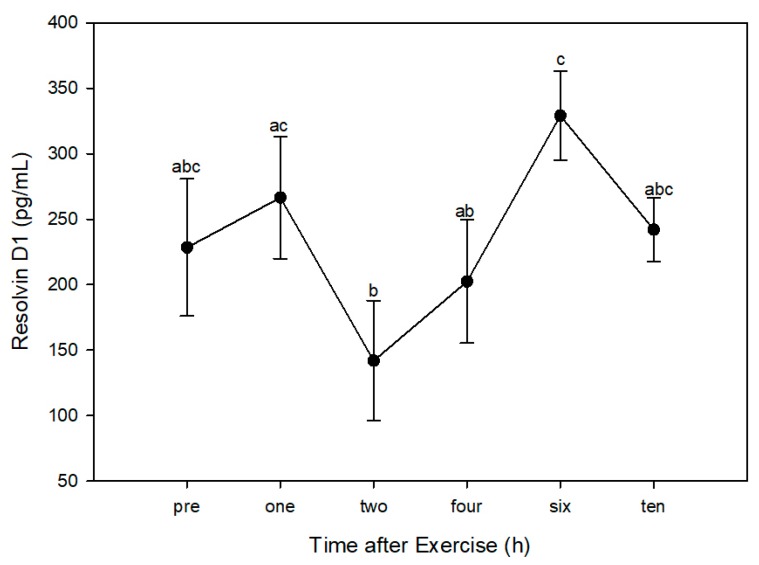
Plasma levels of Resolvin D1 (RvD1) in four dogs prior to (PRE) and 1, 2, 4, 6, and 10 h after a one-hour hunt exercise bout. Time points with different letters indicate significantly different RvD1 concentrations using the “all-pairwise” comparison procedure (*p* < 0.05).

**Figure 3:**
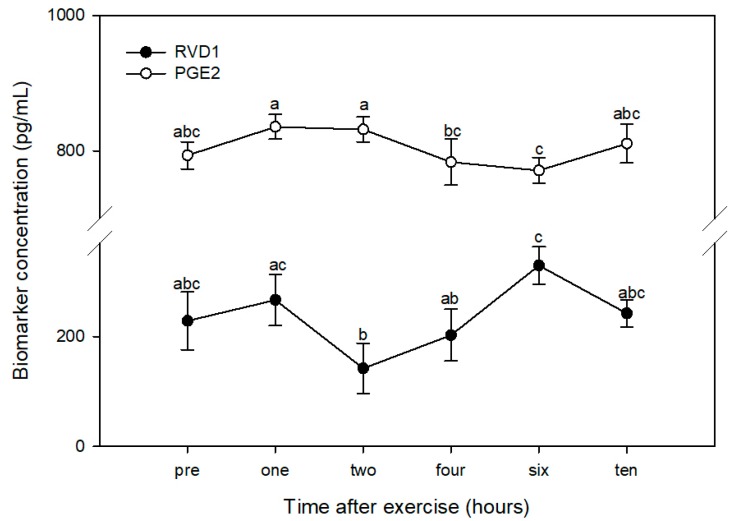
Plasma levels of Resolvin D1 (RVD1) and prostaglandin E_2_ (PGE_2_) in four dogs prior to (PRE) and 1, 2, 4, 6, and 10 h after a one-hour hunt exercise bout. Time points with different letters indicate significantly different concentrations within each biomarker using the “all-pairwise” comparison procedure (*p* < 0.05).

Using pre-exercise values (228.5 ± 52.5 pg/mL) as the control within the post-hoc test, there was a significant increase in RvD1 six hours after exercise (329.2 ± 34.2 pg/mL). Compared with the peak at six hours, RvD1 was significantly lower at two hours (141.9 ± 45.8 pg/mL) and four hours (202.4 ± 47.2 pg/mL) post-exercise. There was also a significant decline in RvD1 between one (266.5 ± 46.6 pg/mL) and two hours post-exercise (Figure 2). RvD1 was at its lowest point at two hours post-exercise, the time at which PGE_2_ was at its peak; similarly, RvD1 was at its peak at six hours post-exercise, when PGE_2_ was at its lowest point (Figure 3). PGE_2_ peaked around one to two hours post-exercise, at which time RvD1 was at its lowest level. As RvD1 began to increase and peaked at six hours post-exercise, PGE_2_ was at its lowest concentration.

### 3.3. IL-1β, TNFα, and NO

There were no significant effects of the exercise on IL-1β, TNFα, or NO (Table 1).

## 4. Discussion

It is well established that animals undergo a wide range of physiological and biochemical adaptations due to exercise, the magnitude of which is related to such factors as previous training and the intensity and duration of the exercise bout [23,24]. The current pilot study provides new information on transient inflammatory consequences of a one-hour hunt exercise in basset hounds. To our knowledge, this is the first study to report a time-course of post-exercise systemic PGE_2_ production in dogs. The duration and intensity of the exercise in this study resulted in a significant increase in PGE_2_ one hour after exercise, consistent with our previous study which showed a significant increase in PGE_2_ one hour after approximately 30 min of exhaustive galloping exercise in horses [4]. Similarly, human athletes experienced a peak in plasma PGE_2_ two hours after a two-hour high-intensity football training bout [12] and one hour after approximately 30 min of exhaustive treadmill running [25]. PGE_2_ is a pro-inflammatory eicosanoid, reviewed in [26], which functions as a systemic pyrogen [27] and contributes to exercise-induced increases in core body temperature [28]. In the cardiac muscle, inducible PGE_2_ (via Cox-2) plays an important role in late-phase ischemic preconditioning, which protects the heart from potentially catastrophic outcomes from ischemia [29]. External ischemic preconditioning is considered to be an effective intervention for improving exercise performance [30,31], suggesting that exercise-mediated elevations in PGE_2_ may contribute to a desirable adaptation of muscle to exercise stress. Indeed, PGE_2_ plays a role in protein synthesis in skeletal muscles following exhaustive eccentric leg exercise in recreationally active human males [32], likely due in part to the stimulatory effect of PGE_2_ on the muscle stem-cell function [31], which is impaired in the presence of cyclooxygenase inhibitors [33,34]. The current study demonstrates that one hour of hunt exercise in basset hounds follows an acute temporal pattern of post-exercise increase in PGE_2_ that is similar to the pattern reported in humans undergoing resistance exercise [19] and in horses undergoing high-intensity exhaustive sprint exercise [4].

A relationship between exercise-induced PGE_2_ and pro-resolving compounds such as RvD1 was observed in LPS-stimulated polymorphonuclear cells isolated from human athletes ([12], reviewed in [18]). In the current pilot study, we observed an inverse relationship between PGE_2_ and RvD1; peak post-exercise PGE_2_ (at one and two hours) coincided with minimum post-exercise RvD1 (at two hours) and minimum post-exercise PGE_2_ (at six hours) occurred at the time of peak RvD1 (at six hours). While this pilot study does not present sufficient evidence for a direct inhibitory effect of PGE_2_ on RvD1 or vice versa, their pro-inflammatory and pro-resolving activities (respectively) support an interpretation of a negative association between these two biomarkers. The peak in RvD1 following resistance exercise in humans immediately precedes a decline in PGE_2_ [reviewed in 18]; the slight difference in temporal accumulation of these compounds compared with the current study may be a reflection of the exercise intensity or species differences. Overall, our pilot study data contribute to a growing body of evidence for antagonistic activities of PGE_2_ and RvD1 during exercise recovery, with the outcome favoring RvD1 and inflammation resolution.

The current study did not observe an effect of hunt exercise on plasma NO concentrations. This is in contrast to what we observed in our previous horse pilot study, where we reported an increase in plasma NO concentration at 30 min, one hour, and two hours after 30 min of exhaustive galloping exercise on a track [4]. Humans also show an increase in salivary NO concentration at one and three hours after both a maximal aerobic test and a high-intensity interval training test on a mechanical ergometer, but not after resistance exercise of swats, leg curls, and stiff exercises [35]. NO is produced during events of oxidative stress and its increase following exercise can be predicted due to its role (together with PGE_2_) in facilitating increases in local blood flow to the exercising skeletal muscles [36,37] and reviewed in [38]. There are variations in NO production depending on exercise intensity [35]; it is possible that the lack of effect observed in the current study resulted from the relatively low intensity of the exercise bout.

Previous research proposed that muscle and connective tissue micro-trauma induced by high-intensity exercise causes the release of pro-inflammatory cytokines, resulting in acute transient inflammation reviewed in [39]. The current pilot study did not detect changes in either IL-1β or TNF-α. Little information is available that clearly describes inflammatory effects of exercise in dogs. Consistent with the current study, Yazwinski et al., [40] found no changes in plasma TNF-α, nor did they find exercise effects on interleukin 6 (IL-6), interleukin 8 (IL-8), or interleukin 15 (IL-15) following a 35 km sled-dog race. Others reported a significant decline in plasma TNF-α in samples obtained from sled dogs after two consecutive days of racing (52–75 km/day) [41], but the precise time after cessation of exercise that the sample was taken is unclear, prohibiting direct comparison to the current study. Horses exercising at low intensity (i.e., endurance exercise of 80–160 km) also did not have measurable increases in IL-1β or TNFα [42].

As this study was designed as a pilot study with a small number of animals, the results reported herein should be interpreted with caution. Furthermore, an unexercised control group was not tested concurrent with the exercised group, preventing us from differentiating contributions of the exercise from those of non-exercise-related factors, such as adrenergic stimulation from psychological stress, which may have contributed to the observed PGE_2_ and RvD1 responses [41]. It is recommended also that future studies employ accelerometers on individual dogs, in order to clearly record distance travelled for each dog within the exercise test. Despite these limitations, the current pilot study provides evidence that a one-hour hunt exercise produces transient inflammation in basset hounds that is characterized by an acute increase in plasma PGE_2_ at one hour post-exercise, falling to a low point at six hours post-exercise. This post-exercise transient pattern of PGE_2_ has an apparent inverse association with a decline and rise of RvD1 at similar time points. Future studies should seek to further understand the distinct and combined roles of PGE_2_ and RvD1 in the adaptation of athletes to exercise.

## 5. Conclusions

This pilot study on four basset hounds demonstrates that one hour of hunt exercise results in transient systemic inflammation characterized by an increase in plasma PGE_2_ at one and two hours post-exercise, followed by an increase in RvD1 at six hours post-exercise. The data can be used to inform future studies with larger numbers of animals to better understand the acute systemic response to moderate-intensity exercise in hunting dogs and its role in the adaptation of animals to exercise stress.

## Figures and Tables

**Table 1:** Plasma concentrations of biomarkers (± standard error of the mean (SEM)) 30 min prior to (PRE) and 1, 2, 4, 6, and 10 h after a one-hour hunt exercise bout in four basset hounds.

Time	PGE_2_ (pg/mL)	RvD1 (pg/mL)	IL-1β (pg/mL)	TNFα (pg/mL)	NO (µg/mL)
PRE	793.5 ± 20.1	228.5 ± 52.5	89.3 ± 6.9	319.7 ± 305.3	7.2 ± 0.8
1	835.6 ± 18.3	266.5 ± 46.6	90.1 ± 3.8	321.4 ± 306.9	7.1 ± 2.3
2	831.8 ± 18.3	141.9 ± 45.8	99.0 ± 5.7	15.1 ± 0.3	5.9 ± 0.2
4	783.6 ± 34.2	202.4 ± 47.2	93.9 ± 7.8	316.5 ± 301.8	5.8 ± 0.9
6	771.3 ± 19.2	329.2 ± 34.2	89.3 ± 6.7	14.4 ± 0.6	7.4 ± 3.5
10	810.8 ± 28.6	242.0 ± 24.4	97.4 ± 6.9	320.7 ± 305.7	7.9 ± 4.3

PGE_2_: prostaglandin E_2_, RvD1: resolvin D1, IL-1β: interleukin 1β, TNFα: tumour necrosis factor α, NO: nitric oxide.
